# Magnetic Resonance Imaging for Translational Research in Oncology

**DOI:** 10.3390/jcm8111883

**Published:** 2019-11-06

**Authors:** Maria Felicia Fiordelisi, Carlo Cavaliere, Luigi Auletta, Luca Basso, Marco Salvatore

**Affiliations:** IRCCS SDN, Via Gianturco 113, 80143 Napoli, Italy; mfiordelisi@sdn-napoli.it (M.F.F.); carlocavaliere1983@yahoo.it (C.C.); lbasso@sdn-napoli.it (L.B.); direzionescientifica@sdn-napoli.it (M.S.)

**Keywords:** translational medicine, preclinical imaging, rodent models, oncology, magnetic resonance imaging

## Abstract

The translation of results from the preclinical to the clinical setting is often anything other than straightforward. Indeed, ideas and even very intriguing results obtained at all levels of preclinical research, i.e., in vitro, on animal models, or even in clinical trials, often require much effort to validate, and sometimes, even useful data are lost or are demonstrated to be inapplicable in the clinic. In vivo, small-animal, preclinical imaging uses almost the same technologies in terms of hardware and software settings as for human patients, and hence, might result in a more rapid translation. In this perspective, magnetic resonance imaging might be the most translatable technique, since only in rare cases does it require the use of contrast agents, and when not, sequences developed in the lab can be readily applied to patients, thanks to their non-invasiveness. The wide range of sequences can give much useful information on the anatomy and pathophysiology of oncologic lesions in different body districts. This review aims to underline the versatility of this imaging technique and its various approaches, reporting the latest preclinical studies on thyroid, breast, and prostate cancers, both on small laboratory animals and on human patients, according to our previous and ongoing research lines.

## 1. Introduction

Translational medicine is an interdisciplinary branch of biomedicine, which aims to translate results from preclinical (in vitro and in vivo) research to the clinical setting, the so-called "from bench to bedside" path [1]. Its main objective is to improve prevention, diagnosis, and treatment strategies for human diseases by filling the gap between the preclinical and the clinical setting. For fruitful translational research, it is fundamental that the discovery process, and the relative experimental design, is immediately aimed at its application in clinical practice [1,2].

In this context, the correct choice of the experimental model and the study design are a fundamental part of the translational plan, and they must be made carefully. In oncology, the use of animal models represents a still-irreplaceable step after in vitro studies. Indeed, they are an essential source of in vivo information, which validates in vitro results and improves their translational value [3,4,5]. Rodents, particularly mice, are the most used in vivo oncological models, thanks to their high genetic homology with humans, their easy genetic manipulability, and their fast reproductive cycle. Hence, their use allows for concluding studies in a relatively short time [3,4,5,6,7].

In vivo preclinical imaging makes possible noninvasive longitudinal studies; thus, achieving a reduction in the biological variability and a substantial decrease in the number of animals while maintaining the statistical power of the data [8,9,10]. Most imaging techniques for small animals are already the same used in clinical settings, i.e., computed tomography (CT), positron emission tomography (PET), high-frequency ultrasonography (HFUS) and magnetic resonance imaging (MRI), and hence, are readily translatable. In oncological research, these imaging modalities are useful in studying drug biodistribution, monitoring treatment response, and identifying new molecular targets and biomarkers for early tumor detection [3,11,12,13,14,15]. Preclinical scanners have to deal with smaller "subjects" with higher metabolic and physiological rates. Hence, dedicated scanners are designed to meet these needs in terms of higher spatial and temporal resolutions but still adopt the same physical and technological principles of their clinical counterparts, which lead to more direct and faster translatability of findings [15].

In this perspective, the MRI can be considered the least invasive and most comprehensive technique, from an anatomo-functional point of view, in the clinical setting. The preclinical MR scanners have to, as mentioned deal with a smaller field of view and higher spatial resolution, assured by the use of higher magnetic fields; e.g., up to 11.4 T. Nonetheless, novel sequences able to show specific pathophysiological features in oncologic disease might be directly translated to the clinic [9,16,17].

This work aimed to review the most recent scientific literature to highlight the translatability of MRI from preclinical animal studies to clinical oncology. In particular, we focused on thyroid, breast, and prostate cancer rodent models, according to our previous and ongoing research lines. Indeed, such histotypes display a significant impact both on clinical oncology and in preclinical research. Moreover, to underline the versatility of the MRI techniques, some preclinical studies on human patients have been reported, as well.

## 2. Animal Models in Oncology

Four broad categories of oncological small animal models are used: xenografts, orthotopic, patient-derived tumor xenografts (PDXs), and genetically engineered mouse models (GEMMs). As for spontaneous human cancer, chemical and radiation-induced murine models have also been developed, and they seem to behave more closely like human tumors [18,19]. Finally, specific considerations will be made about metastatic models. For most animal models, general anesthesia is usually needed, either to eliminate the stress and the pain linked to a surgical procedure, or to obtain immobility of the mouse. The other procedures are usually executable with physical restraint by personnel well-trained in handling animals [20].

### 2.1. Xenograft and Orthotopic Models

Subcutaneous and orthotopic xenograft models are based on cancer cell lines directly derived from human tumors. Such cell lines belong from primary, lymph nodes, or metastatic tumors; they are immortalized and usually well-defined from the genetic point of view [21,22]. Hence, they allow extremely reproducible experiments for growth rate, metastatic potential, histopathologic homogeneity, and biological behavior. For their growth in the host species, i.e., the mouse, the host itself has to be immunocompromised [3,22]. Depending on the cell line, various degrees of immune system “knock-out” might be necessary [23,24]. In general, these models summarize well, as mentioned, the histopathological features present in the original tumoral tissue, but the use of immunodeficient mice may hinder the study of the tumor–host immune interaction. Subcutaneous xenografts are more easily reproducible compared to orthotopic implantation, which usually requires invasive surgical procedures. However, xenografts are not representative of the original tumor in its native environment [21,25]. On the other hand, the use of orthotopic models reestablishes the interactions between the tumor and its origin organ and might recapitulate the metastatic behavior with sufficient penetrance and reproducibility. Nonetheless, a limit of orthotopic models, besides the lack of interaction with the host’s immune system, is that they do not allow modeling the pre-neoplastic process since the cell line already has completely tumorous genetic and biological behavior [18,25,26]. Depending on the murine strain selected, these models might be less expensive than GEMMs [25,26]. These animal models, for their reproducibility, seem to be ideal for preclinical and translational studies concerning therapies for tumor growth and angiogenesis inhibition. Furthermore, they are useful for studying the pathogenetic aspects of cancer vascular invasion and metastasis [26,27,28].

Both xenograft and orthotopic models are used to replicate thyroid cancer. In the xenograft models, selected cell lines are injected into the subcutaneous tissue of immunodeficient mice. The selection of the appropriate cell line is important not only to fulfill the experimental aims but also because it has been demonstrated that among 40 thyroid cell lines, almost 40% are cross-contaminated or misidentified [29,30]. Hence, genetic evaluation of the chosen cell lines should always be performed [30,31]. The orthotopic model is more complex and requires microsurgery to inject tumor cells directly into the murine thyroid gland [25,26,32]. Recently, an HFUS-guided injection procedure has been presented and resulted in less invasiveness compared to the standard surgical procedure [33]. Such a procedure was easily reproducible for the orthotopic implantation of thyroid carcinoma cells and allowed for continuous monitoring of the disease, for a more extended period and with less contamination of other neck structures by the carcinomatous cells compared to the surgical approach [33]. In thyroid cancer research, the orthotopic model allows the understanding of the molecular and cellular mechanisms of thyroid cancer pathogenesis, improving the evaluation of new therapeutic compounds, and testing the effects of therapeutic interventions on both the primary tumor and metastases [34,35].

The xenograft and orthotopic mice models are widely used for investigating breast cancer. These are commonly produced by injecting subcutaneous tumor cells in the flank or orthotopically in the mammary fat pad of immunocompromised mice. The xenograft models allow one to study, in vivo, the tumor environment, and tumor growth, but the absence of an intact immune system profoundly affects tumor development, in particular, its early stages, and the progression of the metastatic process. Orthotopic models of breast cancer provide a more favorable microenvironment, but there are critical differences between human and mouse mammary stroma [36]. Commonly, these models involve athymic nude, severe combined immunodeficiency (SCID) and non-obese diabetic (NOD) SCID mice, which show genetic and immunological differences that mainly influence the induction and dissemination of metastases to organs. Indeed, natural killer (NK) cells and the remaining innate immune cells in nude and SCID mice probably contribute to the reduction of tumor engraftment, growth, and metastases in these models. On the other hand, NOD SCID and particularly NOD SCID gamma (NSG) represent a better background to study breast cancer metastases due to the lack of NK cells, and thus they could be considered ideal for the study of anti-metastatic treatments [37,38,39].

Prostatic cancer models are usually xenografts, in which tumor cells are inoculated subcutaneously, and the cellular lines mostly used are either androgen-independent (PC-3 and DU-145) or androgen-responsive (LNCaP) cells. Such a set of cell lines reproduces various features of the naturally occurring disease [40,41]. Orthotopic prostate tumor models are relevant to study the growth and metastasis of prostate cancer. Generally, tumor cells are injected into the prostates of immunodeficient mice; however, there is a low incidence of tumor formation due to technical difficulties that can be overcome by intratesticular inoculation [41,42,43].

The xenograft models of colon cancer are rarely used [44]. The orthotopic model represents an accurate reproduction of the human pathology and can be generated by the implantation of tumor cells, via a surgical celiotomy, under the cecal serosa. Alternatively, a model generated by trans-anal rectal injection has been developed, and it may allow a more accurate investigation of the inflammatory and immune responses, without the influence of previously-used abdominal surgery. The orthotopic colon cancer model is useful in inducing local tumor growth but is limited by a low rate of lymph node and hepatic metastases [45,46].

### 2.2. Patient-Derived Xenografts (PDX)

The PDX models are based on portions of primary tumors, are collected by biopsy or surgical excisions, and are transferred from the patient in immunodeficient mice by subcutaneous or orthotopic implantation. Since these models are derived directly from the patient’s tumor, they have a great translational potential for the study and development of anticancer therapies in the perspective of personalized medicine. The main advantages of PDX are that they duplicate precisely, the original clinical tumor compared to long-established cell lines, and hence, they preserve the molecular, genetic, and histological heterogeneity of naturally occurring cancers [47,48,49]. These models have been shown to have a predictive value in the clinical outcome and are useful for studying novel or alternative drugs, in order to identify biomarkers for early predictive responses to the treatment, and thus, to guide therapeutic approaches in clinical patients. Furthermore, when this model is generated by an orthotopic implant, it often reproduces, as mentioned, the same metastatic process of the human disease. All these features give to PDX models, the ability to study metastases and the genetic evolution of a tumor [47,48,49]. There are some challenges concerning PDX models. Occasionally, to obtain a reasonable growth rate, they are propagated between different hosts; i.e., the graft is removed from one mouse and implanted in another one. During this passage, the stromal components of the mouse may become dominant over the human one; hence, limiting the translatability of results of the tested therapies. Moreover, the interaction of the tumor with the immunocompromised hosts does not allow studying the interaction with the immune system during the anticancer treatment, eventually hiding crucial therapeutic mechanisms [47,49,50]. Last but not least, the limitation of this model is linked to long bureaucratic waiting periods when obtaining the necessary authorization for animal experimentation. Only big governmental projects might be prepared from the perspective of a truly personalized medicine approach [48].

Concerning thyroid cancer, PDX mouse models accurately reproduce the tumor microenvironment and the biochemical interaction between tumor cells and stromal components, as previously mentioned [47]. However, particularly for medullary thyroid carcinomas, there are serious difficulties in generating a murine PDX model due to the slow tumor growth [51].

The PDX models of human breast cancer are gaining high relevance for preclinical evaluations of experimental therapeutics. In general, cells derived from patients are transplanted into a mammary fat pad of immunocompromised mice, particularly in NSG mice that are commonly used and better suited for PDX generation for their high engraftment rates [52,53].

The PDX models are used to highlight various aspects of prostate cancer in preclinical research. In particular, they are used to test individual responses to therapy, to clarify the angiogenetic pathways, and to identify new antimetastatic treatments. The tumor/human stroma interaction can support tumor growth and improve the onset of metastases resembling the naturally occurring disease. However, there are several issues during the orthotopic implantation, related to the size of the fragment and the choice of the implantation site, which have led to lower availability of PDX models of prostate cancer compared to other histotypes [41,54].

The PDX of colon cancer is generally generated using cultured primary cells derived from a patient’s colon tumor, which are injected orthotopically into the cecal serosa. The direct transplantation of colon tumor tissue is being discarded, due to the bacterial contamination and the consequent septic shock in the immunocompromised host. Moreover, a significant amount of tissue is required, which can hardly be replaced in the event of engraftment failure. Nonetheless, PDX models of colon cancer allow the monitoring of tumor growth, metastatic evolution, and the pharmacological responses of individual patients [55,56].

### 2.3. Genetically Engineered Mouse Models (GEMMs)

The GEMMs play a significant role in cancer research, and they can be considered the most advanced animal models recapitulating human pathology [21,57]. The GEMMs develop tumors in situ, according to the gene(s) activated and/or deleted in immunocompetent, genetically manipulated, mice; the tumor growth in such models can be either spontaneous or molecularly-induced. These characteristics are extremely useful in studying the complex processes of carcinogenesis and also allow evaluating the interactions between tumor cells and the immune system in a more “natural” microenvironment [18,21]. These models better reflect human disease in terms of biological behavior, histology, and genetic accuracy, but they also show some disadvantages, such as variable penetrance, heterogeneous growth, and tumor latency, and they are expensive [4,13,15,21,57].

Transgenic thyroid cancer mouse models are widely used to study the numerous mutations already systematically identified in human specimens [25,32]. The GEMMs models represent a way to study carcinogenesis, tumor progression, and metastatization, and hence, responses to therapeutics. The use of these models of animals with intact immune systems, as well as the ability to histologically reproduce human tumors, allows the evaluation of different drugs at different stages of the disease during tumoral development. However, these models are not useful for evaluating antimetastatic therapies since they do not always reproduce the specific pathways of human disease [32,57]. They are also useful for the study of unconventional therapies, such as micro-RNA, small interfering-RNA, or immunotherapies, during the early events of tumor transformation and progression, and as a promising approach to customizing anticancer therapy [58].

The GEMMs models of breast cancer display many features of the human pathology, although they do not entirely recapitulate all its aspects, due to the complexity and heterogeneity of breast cancers. However, these models are invaluable for investigating the biology and pathogenesis of breast cancer, and they are especially helpful for elucidating the mechanisms that regulate the initiation and progression of this disease, as well as for understanding the genes involved in these phases [36,59]. Transgenic mouse models of breast cancer are developed in immunocompetent hosts, and therefore, they allow evaluating the cancer/host immune interaction. Some of these models spontaneously metastasize to the lungs, whereas the incidence of bone metastases is very low. Furthermore, there are some selective tissue promoters, such as the whey acidic protein and the mouse mammary tumor virus, which may be directed to the oncogenic expression of the mammary gland [36,60].

In the study of prostate cancer, GEMMs reproduce, in depth, its different stages, and their use has provided valuable information on tumor initiation and progression. These transgenic models have been divided into different categories. The first generation of GEMMs utilized probasin to produce the probasin-large T antigen transgenic mouse (LADY) and the transgenic adenocarcinoma of the mouse prostate (TRAMP) models, which both show an aggressive phenotype, often with metastatic bone involvement. On the other hand, the second generation GEMMs have integrated the single molecular changes observed in human diseases, such as the loss of phosphatase and TENsin homolog (PTEN) and Myc over-expression, but they seem to be unable to reproduce the different stages of prostate cancer [54,61].

A variety of GEMMs have been generated as models for colon cancer to understand how common, coexisting mutations cooperate in a natural environment. These transgenic models are useful for studying pathogenesis and testing potential therapeutic agents, although they do not show phenotypes similar to human diseases. Moreover, these models are not practical for studying colon cancer metastases [44,62].

### 2.4. Chemical and Radiation-Induced Models

These animal models are valuable tools to study mechanisms underlying human carcinogenesis. From that perspective, these murine models play an essential role in the interpretation of epidemiological observations: to assess the natural history, mechanism, and modifying factors of cancer development. Furthermore, these models allow evaluating the growth and histologic characteristics of the primary tumor and metastases. Nonetheless, they show high variability in terms of the time of onset and prevalence, as well as a long latency in metastatization; thus, influencing the number of animals required for a study [18,19].

Chemical agents or physical agonists and radiation, as well as the administration of known carcinogenic substances, have been used to induce thyroid cancer in mice. Mainly these are goitrogens that decrease thyroid hormone levels, resulting in alterations that then induce thyroid tumorigenesis. Furthermore, it is known that ionizing radiation is a determinant risk factor for thyroid cancer, but it is difficult to obtain a systematic and rapid model [63,64].

Radiation-induced mouse models and chemical carcinogenesis have been developed for the study of breast cancer. Through these models, the natural development of breast cancer linked to exposure to carcinogens has been evaluated. Radiation-induced models are relevant since radiation exposure is one of the epidemiologically-proven etiological factors of human breast cancer [65]. Moreover, chemical-induced breast cancer can be induced in some strains of rats by administering dimethylbenzanthracene and *N*-methyl-*N*-nitrosourea (MNU) [19,66].

Prostate cancer rodent models are rarely used. In particular, the most common is the chemically-induced, following the administration of methyl nitrosourea and testosterone in rats; these models infrequently recapitulate metastatization, and only will to the lymph nodes and lungs [66].

Chemically-induced colorectal cancer models were developed in mice following exposure to carcinogens, such as dimethylhydrazine, *N*-methyl-*N*-nitro-*N*-nitrosoguanidine, and MNU. The incidence depends on the dosage, duration, and frequency of administration; the route of administration; i.e., oral, subcutaneous, or intrarectal; and the timing of administration. These models are useful for studying the influence of diet on tumor development. However, the occurrence of cancer is low, making the use of a large number of animals necessary, and the metastatic phase is prolonged and infrequent [67].

### 2.5. Metastatic Models

The development of animal models of metastases is useful for the evaluation of new therapies that can prevent the metastatic phase or arrest the growth of metastasis already formed, and to identify new molecular targets. There are numerous approaches for the development of such models, including experimental metastases, spontaneous metastases, and the GEMMs’ metastatic approach models [60,68]. Experimental metastasis models are established by direct injection of the selected cell line into the bloodstream through the tail vein or via intracardiac injection. Such an approach allows the diffusion of metastases to various organs, including the lungs, bones, brain, liver, and lymph nodes. The time-course is generally short, and the biology of metastases results is reproducible, but the injection site influences the target organ, and above all, this approach bypasses the early phases of the metastatic cascade [68]. The spontaneous metastasis model was derived from the orthotopic model, and more closely resembles the human pathology, providing the cascade of metastatic processes with adequate reproducibility [68,69]. The GEMMs’ metastasis models show different degrees of penetrance and latency, and therefore, allow evaluating the metastatic process in a heterogeneous genetic background. Indeed, the activation or the loss of a gene may not replicate completely natural metastasis. Besides, the penetrance for the metastatic process is often low. Finally, long latency times and difficult recognition of metastases have been reported [68].

In thyroid cancer research, all the models above of metastatization are used. The orthotopic model is essential for evaluating the molecular mechanisms preceding metastatization. Often, the local invasion of neck’s structures by the primary tumor, i.e., large vessels, the esophagus, and the trachea with consequent respiratory insufficiency or inability to feed, do not allow enough time to study metastases. On the other hand, most transgenic mouse models develop a limited number of spontaneous lung metastases, which are only detected post-mortem at the end of the experiment. Tail vein and intracardiac injections of thyroid cancer cell lines are used, as described in [32,70,71].

The orthotopic injection of cells into the mammary glands offers a comprehensive model of the metastatic process. A robust breast cancer metastasis model is obtained using immunocompromised NSG mice that facilitate understanding the mechanism underlying breast cancer metastatization [60,72,73]. Usually, this model causes a low incidence of bone metastasis, frequent in human beings. In any case, metastatization in mice models of breast cancer is strictly linked to the cell line used. Some transgenic models of breast cancer spontaneously metastasize to the lungs, but there is a very low incidence of bone metastases, probably due to the rapid progression of primary tumors [60].

Human prostate cancer cell lines have been orthotopically implanted into mice to study the different stages of cancer progression up to metastatization. This model also reproduces bone metastases, which, however, show a low incidence. Furthermore, the so-called mouse prostate reconstitution model is considered a valid approach for the development of bone micro-metastases. This model combines human neoplastic and non-neoplastic prostate cells with the murine urogenital sinus mesenchyme, and thus, the cells are implanted under the renal capsule of an immunodeficient mouse. This method allows for exploring the genetic pathogenesis of prostate cancer, but technical complexity often limits its use [60]. Most transgenic models of prostate cancer do not develop metastases or show a very low incidence, especially of bone metastases, often due to the rapid progressions of the primary tumors [60,66].

The metastatic colon cancer model is developed by orthotopic microinjection of human colon cancer cells directly in the subserosa of the cecum, via surgical celiotomy, or the injection into the portal vein. These traditional methods reproduce metastatic colonization of the liver and lungs and facilitate the study of the metastatic cascade that follows both qualitatively and quantitatively, the natural course. A noninvasive trans-anal model has been developed, with a trans-anal rectal injection of colon cancer cells after disruption of the mucosa with irritant agents administered via enema. This model allows assessing essential aspects of the development of metastases, without the negative influence of the surgical intervention. Transgenic metastasis models of colorectal cancer offer reproducible information on tumor onset and in the early phases of the metastatic process but have a low metastatic rate and limited dissemination to the target organs [74,75,76,77,78].

## 3. In Vivo Imaging

In clinical practice, CT, PET, ultrasonography, and MRI are essential for the diagnoses and monitoring of neoplastic diseases. In preclinical experimental models, these modalities provide functional and metabolic imaging and pathophysiological information beyond morphology, as well as the ability to study molecular events. In both clinical and preclinical areas, imaging modalities contribute to the oncology field by being reliable indicators for early tumoral responses to therapy, and they can guide effective therapies, or stratify patients [79,80]. General anesthesia is usually needed, in the case of imaging, to obtain immobility of the mouse, and to abolish the stress due to the loud noise, especially that produced by MRI [20].

### 3.1. Computed Tomography (CT)

In the clinical field, CT imaging has extensive applications in inflammation, angiography, cancer detection, and the evaluation of bone regeneration or toxicities. It provides useful anatomical details, but exposure to high radiation often limits the number of scans performed in the same patient. In preclinical research, this methodology, besides being particularly useful for the assessment of skeletal and lung abnormalities and cardiac function, is considered a robust technique for the quantitative evaluation of angiogenesis associated with solid tumors. The small animal CT has a high spatial resolution (up to 10 µm, in vivo) and relatively short imaging times. However, the employment of clinically-used contrast agents limits the repeated imaging in the same animal, in particular, due to the iodinated contrast, which is rapidly cleared from the blood, and therefore, should be administered repeatedly. Moreover, high dosages of this agent may result in nephrotoxicity, and its use may produce an ionization effect, resulting in radiation damage through reactive oxygen species [8,17,81,82,83,84].

### 3.2. Positron Emission Tomography (PET) 

The PET has become a powerful tool for clinical diagnosis and preclinical research, and it plays an essential role in oncological research in monitoring metabolism, gene expression, cell proliferation, angiogenesis, hypoxia, and apoptosis, as well as for drug development [85]. Indeed, this modality, in the clinical setting, is used for tumor staging, to evaluate tumor response to therapies through the use of an radiolabeled imaging agent, such as [18F]-2-fluoro-2-deoxy-glucose [18F]-FDG) for glucose metabolism or [18F]-fluoro-3’-deoxy-3’-L-fluorothymidine [18F]-FLT) for cell proliferation [86]. The PET also has a wide range of applications in preclinical research; for example, it allows investigating physiological and molecular mechanisms of human diseases, and evaluating novel radiolabeled imaging agents, and the biodistribution and the efficacy of new drugs in suitable animal models [87]. As for clinical scanning, preclinical PET devices provide excellent sensitivity (from 2% to 7%, depending on the energy window) and spatial resolution (1.2 mm, on average), but the main disadvantages are the lack of an anatomical parameter, usually overcome by associating a CT scan or MR scans. Moreover, the short half-life of most radioisotopes requires strict coordination between radiotracer production, delivery, and use in small animal models [83,84].

### 3.3. Single-Photon Emission Computed Tomography (SPECT)

Single-photon emission computed tomography (SPECT) is a functional, nuclear medicine technique based on the detection of gamma photons emitted by a radionuclide during its decay. SPECT employs a gamma camera, composed of detector crystals and a lead or tungsten collimator with multiple elongated holes—pinholes, which rotates around the subject and acquires multiple cross-sectional images. This technique is similar to PET, and it is widely employed in clinical routines, but it is less sensitive than PET. Indeed, the spatial resolution of clinical SPECT (8–10 mm) is lower than that of clinical PET (4–6 mm) [17,83]. The main advantage is the availability of several radiotracers (^99m^Tc, ^67^Ga, ^111^In, and ^123^I), which have relatively long half-lives compared to most PET radiotracers. In oncological research, SPECT tracers targeting angiogenesis, hypoxia, acidosis, and metabolic activity have been developed and applied [82]. In preclinical investigations, micro-SPECT has a higher spatial resolution (0.35–0.7 mm), compared to micro-PET, and as mentioned, the longer half-life of SPECT tracers is advantageous in terms of production and transport. On the other hand, in longitudinal studies, researchers have to wait for complete decay accordingly. The SPECT’s main application is the study of the biodistribution and kinetics of novel radiopharmaceuticals. Indeed, preclinical PET scanners and radiopharmaceuticals showed a stronger ability to study tracers’ kinetics compared to SPECT counterparts [82,83].

### 3.4. High-Frequency Ultrasonography (HFUS)

Compared to other molecular imaging modalities, HFUS represents a noninvasive, more cost-effective modality, with excellent temporal and spatial resolutions (up to 30 µm axial resolution and 70 µm lateral resolution). It shows the ability to obtain real-time anatomo-functional data rapidly. Moreover, this modality can be implemented by using microbubbles as a molecular-target contrast agent to enhance image quality and specificity. Ultrasound imaging is clinically used for routine screening examinations on breast, abdomen, neck, and other body districts, as well as for therapy monitoring. In this setting, over the last few years, the sensitivities and specificities of ultrasound devices to detect microbubbles have progressively improved. Indeed, beyond the frequent use of color or power dopplers, non-targeted microbubbles are used as intravascular contrast agents improving the detection and characterization of cancerous lesions, inflammatory processes, and cardiovascular pathologies [83,84,88]. In preclinical investigations, HFUS can be applied to monitor tumor growth and vasculature development, and in combination with contrast-enhanced microbubbles agents (CEMAs), is used to assess tumor angiogenesis, inflammation, and therapeutic effects. For example, the vascular endothelial growth factor receptor 2 (VEGFR2), which is a molecular marker of angiogenesis and is overexpressed on tumor vascular endothelial cells, is widely used in preclinical cancer research as a marker of therapy responsiveness [88,89,90,91,92]. Therefore, this modality could help enhance the translation of antiangiogenic agents and contribute positively to human patients’ treatments [93].

### 3.5. Magnetic Resonance Imaging (MRI)

The MRI is a noninvasive imaging modality without ionizing radiation, which provides morphological images with excellent soft-tissue contrast and high spatial resolution. The MRI is one technique with multiple possible outcomes; indeed, depending on the sequences, it allows studying, other than the merely displaying the anatomy, of various aspects of the same lesion. In particular, diffusion of water molecules can be studied with diffusion-weighted sequences (DWI); spectroscopy allows detecting and quantifying small amounts of known molecules; angiographic sequences (like time-of-flight or phase contrast angiography) allow evaluating large blood vessels, even without the need of contrast agents, and so on [17,82,83,84]. The MRI has also proven to be useful in preclinical research, allowing the evaluation of biological processes at the molecular level in in vivo rodent models of diseases. The MRI needs complete immobility of the subject since even the respiratory and cardiac motions can induce substantial artifacts, reducing the quality and the information available from images; hence, requiring the use of respiratory gating to minimize the former effect [93].

The use of anesthesia may minimize animal motion, but some anesthetic agents may alter animal physiology, and thereby, potentially affect the biological processes under investigation [94]. Pre-anesthesia fasting times, the anesthetics, and their dose, may all impact biological processes. Pre-anesthetic fasting in mice is generally considered unnecessary, and prolonged fasting can cause hypoglycemia. However, in imaging studies, a full stomach may interfere with adjacent structures. From the metabolic point of view, fasting may display profound effects on glucose metabolism, and hence, for example, it may significantly influence the results of FDG-PET studies. Body temperature should be monitored and kept within thermoneutrality, since anesthesia induces more or less marked hypothermia, which can also negatively affect the quality of some molecular imaging procedures. The choice of the anesthetic protocol is paramount for particular MRI sequences as well. Indeed, anesthetics may display a significant impact on vascular tone and hemodynamics, masking or altering critical experimentally-related results. Nowadays, great drug selection is available, and the choice, as mentioned, should be in accordance to the specific experimental aims. Nonetheless, an ideal anesthetic agent should be easy to administer, produce a rapid and adequate immobilization, have limited side effects, and be reversible and safe for the animals [20,94].

On the other hand, significant technical challenges exist in the transfer of preclinical MRI sequences to the clinic, due to the higher strength of the magnetic fields used in preclinical settings. However, preclinical systems often lack the wide selection of coils available for medical use, and hence, they require the development of specific coils. Therefore, preclinical MRI is essential for the development and validation of novel techniques, but these technical and biological challenges can partly hinder translation [93,95].

### 3.6. Multimodality Imaging

The combination of different imaging methods, the so-called multimodal imaging approach, offers the opportunity to correlate the most advantageous capabilities of each method and provide complementary information while compensating for their limitations. In preclinical research, all possible combinations of molecular imaging techniques are usually employed. In human medicine, the first example is the PET/CT combination, which offers highly specific functional information, with an excellent anatomic co-registration. Such a system is used in preclinical imaging as well. Another fascinating combination is the PET/MRI, which has been used in the last few years, in both clinical and preclinical settings, even if with some critical technical drawbacks. In any case, the multimodal approach should offer an excellent anatomical resolution with functional information, allowing for a multiparametric evaluation within a single study [8,9,83,96,97,98,99,100].

## 4. Magnetic Resonance Imaging Sequences in the Translational Context

The MRI is one of the primary preclinical and clinical imaging modalities, ideal for non-invasive longitudinal studies and useful for monitoring multiple parameters. In the oncological field, MRI is suitable for the definition of lesions with a high spatial resolution and anatomical detail, allowing the identification of primary tumors and metastatic disease. Moreover, it is also useful for the evaluation of quantitative characteristics that provide physiological, biochemical, and molecular details able to predict the biological behavior of cancer [9,32,101]. Various MRI sequences have been successfully developed by preclinical research and translated into clinical applications, as described below. Indeed, the preclinical MRI scanners can be easily considered a translational platform, since both preclinical and clinical scanners work with the same hardware settings and with almost identical sequences [9,102]. However, it is always necessary to establish the reproducibility of the technique chosen before being introduced in the clinic.

As mentioned, the MRI offers the ability to evaluate numerous tumors’ biological properties, such as angiogenesis, perfusion, pH, hypoxia, metabolism, and macromolecular content. Such biological features are used as indicators in preclinical research to monitor a cancer’s response to therapy [93,95]. Moreover, the complementary nature of the information accessible, applying different MRI sequences in the same subject, can undoubtedly help cancer research; clinical management as well [101].

### 4.1. T1 and T2 Weighted Sequences

Image contrast in MRI acquisition is determined by tissue properties, sequence type, and sequence parameters. Several relaxation constants are used to describe the decay and recovery of the MR signal; in particular, the two most commonly used parameters are the T1 and T2, the so-called spin-lattice relaxation time (T1) and spin-spin relaxation time (T2), which are both weighted. Based on the T1 and T2 weighting, many sequences have been developed [103,104,105]. In clinical applications, the T1 and T2-weighted sequences are the standard for anatomical imaging, and they are useful in the oncology field, for the detection and evaluation of macroscopic changes in the lesion, and staging. In addition, exogenous contrast agents, such as gadolinium, manganese and iron-based contrast agents, can be applied to enhance the contrast between tissues or organs [104,106]. In preclinical studies, T1 and T2-weighted sequences are used for anatomical acquisition, with the employment of contrast agents as well [107].

#### 4.1.1. Thyroid

In thyroid cancer, MRI texture analysis derived from conventional sequences reflects different histopathologic features and represents a possible association that can be used as a prognostic biomarker. Thirteen patients with histopathologically-confirmed thyroid carcinoma were enrolled and subjected to MRI acquisitions. The T1-precontrast and T2-weighted images were analyzed; overall, 279 texture features for each sequence were examined and correlated to the histopathological parameters Ki67 and p53, which are considered prognostic biomarkers. Several significant correlations were identified; indeed, sum square average features derived from T1-weighted images and entropy-based features derived from T2-weighted images are associated with p53 count in thyroid cancer. Similarly, different texture features derived from T1- and T2-weighted images showed associations with the Ki67 index. Hence, this relatively simple MRI technique, combined with texture analysis, does not replace histopathological examinations, but may be a novel, noninvasive modality for further characterizing thyroid cancer in clinical oncology [108].

The effects of the tyrosine kinase inhibitor gefitinib on MRI parameters, and the ability of such parameters to help schedule chemotherapy, were tested in a murine model of breast cancer. In this study, a xenograft model with BT474 cells (ductal carcinoma) was induced, and mice were treated with gefitinib either daily for ten days or “pulsed” for two consecutive days (higher dose for two administrations). The MRI acquisitions were performed at 2-day intervals over two weeks, and T1 and T2 weighted sequences were used to follow-up tumor volume. Moreover, diffusion and DCE MRI were performed, and relative results were reported in the respective paragraphs. Treatment with gefitinib resulted in significant tumor growth inhibition, both with pulsed and daily treatment. Hence, T1 and T2 proved to help evaluate growth inhibition but did not give any further information on which therapeutic protocol gave the best results [109].

#### 4.1.2. Breast

In a preliminary study on breast cancer, the MRI texture analysis was used to analyze the correlation between textural features and tumor volume, and to distinguish the underlying molecular subtypes luminal A and B. Patients with histopathologically-proven, invasive, ductal breast cancer were selected. The first frame of T1 and T2-weighted sequences pre-contrast was acquired and followed by the administration of a gadolinium-based contrast agent. The data analyzed mainly in the pre-contrast images showed that luminal A and B types had different textural features. Luminal B types of cancer have, in fact, a more heterogeneous appearance in MR images compared to luminal A types. These subtypes also showed a difference in tumor volume, with luminal B types showing a larger volume than luminal A types. The MRI texture analysis, combining information from both T1 and T2-weighted images, may provide further information on the biological aggressiveness of breast tumors that may improve therapeutic efficacy and management [110].

#### 4.1.3. Prostate

Anatomical MRI sequences may improve the accuracy of evaluating prostate volume, especially in subjects under hormonal treatment. The T2-weighted MRI was performed to evaluate the normal prostate volume in male C57/BL6 mice. The mice underwent castration, and they were repeatedly imaged to follow the castration-induced regression of the prostate. In addition to the T2-weighted sequence, a chemical shift-selective sequence (CHESS) was performed to suppress abdominal fat signal, and hence, to improve prostate distinction. Forty days after castration, each mouse was treated daily with the androgen dihydrotestosterone (DHT) subcutaneously (s.c.) to induced prostate re-growth. These sequences, in particular, the CHESS, allowed good discrimination of both the prostate margins and the ventral lobe from the dorsal and lateral lobes. Hence, this approach may improve the ability to differentiate the prostate from surrounding tissues and to better visualize the boundaries of the organ, even in human patients [111].

### 4.2. Dynamic Contrast-Enhanced

The most common MRI methods available to quantify perfusion, hemodynamic, and vascular properties of a tissue, are the dynamic contrast-enhanced (DCE) MRI and the arterial spin labeling (ASL) methods [93]. The DCE MRI represents an indirect measure of angiogenesis that evaluates the dynamic passage of a contrast agent through the tumor vessels to derive pharmacokinetic properties, measuring the contrast agent extravasation rate (Ktrans) and volume fraction (ve) of the extracellular extravascular space (EES), as thoroughly described elsewhere [93,112,113,114]. Such methods have been used to monitor perfusion, as a marker of responsiveness to antiangiogenic treatments, both in preclinical and clinical trials, in terms of either efficacy or early identification of treatment failure [14,99,115,116,117,118,119,120]. Therefore, the clinical ability of this method is remarkable not only for the functional information on the tumor vascularization pattern but also for the chemotherapy planning, monitoring response, and implementing a new line of therapies [121].

#### 4.2.1. Breast

In the preclinical model of ductal breast cancer with BT474 cells previously described (see 4.1 T1 and T2 Weighted Sequences, [109]), the DCE was also performed at 2-day intervals over two weeks. Transendothelial permeability (Kps), and fractional plasma volume (fPV) were measured. Tumor Kps decreased with pulsed treatment but then rebounded and increased with daily treatment. Tumor fPV increased in both treated groups, subsequently decreasing with pulsed treatment. Therefore, such quantitative MRI parameters may provide a sensitive measure to distinguish treatment regimens, and they might be useful for determining correct treatment scheduling, and hence, enhance chemotherapeutic efficacy [109].

In a preliminary study of breast cancer in human patients, the predictive ability of MRI was demonstrated using the DCE or DWI methods to predict tumor response following neoadjuvant therapy (NAT). This model integrated the heterogeneity of MRI-derived parameters (i.e., efflux rate constant—*Kep*, and ADC, as further described in the respective paragraph) with the hormone receptor status (i.e., estrogen receptor—ER, progesterone receptor—PR, and human epidermal growth factor receptor 2—HER2) and clinical data which were obtained before and after the first cycle of NAT. Thirty-three breast cancer patients underwent neoadjuvant chemotherapy, and therefore, were scanned for DW and DCE-MRI in the following three time-points: before NAT, after the first cycle of NAT, and after all NAT cycles. The median number of treatment cycles was 14 cycles. After completion of NAT, the pathological complete response (pCR) and nonresponse (non-pCR) were determined at the time of surgery. Twelve patients exhibited a pCR, and twenty-one patients were non-pCR. For patients with pCRs, the mean Kep had decreased between the pre and post-first NAT cycles, while for a non-pCR patient, Kep increased. Measurements after all NAT cycles were not obtainable in pCR patients since there was no residual tumor identifiable in the MRI scan. The immunohistochemical evaluation of hormone receptor status has defined the molecular subtypes of breast cancer for all patients; indeed, among the thirty-three patients, nine, six, eight, and ten patients are classified into luminal A, B, HER2, and basal subtypes, respectively. The MRI approach in question seems to accurately predict a treatment response right after a single cycle of therapy. Moreover, this method may improve the accuracy of evaluating a tumor’s response to NAT, showing a higher predictive power than models based on tumor size changes, and it may be used as a short-term surrogate marker of outcome in breast cancer patients [122].

#### 4.2.2. Prostate

Male BALB/c nude mice were implanted subcutaneously with the human-derived, androgen-sensitive CWR22 cell line, to evaluate the predictive capacity of DCE in a prostate cancer xenograft mouse model. These mice received combinations of androgen-deprivation therapy, obtained by surgical castration and/or radiotherapy. The MRI sequences applied were DCE and DWI, which will be discussed in the dedicated paragraph, and they were performed pre-radiation, and on day one and nine; the ADC value, the *K^trans^,* tumor volume, and PSA were used to measure the therapeutic response. The *K^trans^* and PSA showed a high level of correlation with treatment response, and thus, this parameter might be included in the evaluation of treatment responses in prostate cancer patients [123].

### 4.3. Arterial Spin Labeling

As mentioned, the ASL is a noninvasive and quantitative technique that allows perfusion measurement without requiring the administration of a contrast agent. This technique uses arterial blood water as an endogenous diffusible tracer by “labeling” it; i.e., by inverting the magnetization of the blood with radiofrequency pulses. As a result, studies can be repeated in the same subject over time [93,101,124,125,126,127]. Measurement of tumor blood flow with this technique is strongly helpful for tumor grading and evaluation of anticancer treatment [124,126]. The ASL is widely used in the preclinical and clinical fields, but in the latter case, it is still an emerging technique and has not yet replaced more invasive procedures, such as contrast-enhanced MRI, probably due to the complexity of the method and the relatively high sensitivity to motion artifacts [124].

#### Breast

ASL might gain a significant impact on the diagnosis and therapy management of breast cancer, thanks to its ability to quantify perfusion without the use of contrast agents, as examined in a pilot study. Quantification of perfusion of normal fibroglandular tissue and breast cancer using a flow-sensitive, alternating-inversion, recovery-balanced, steady-state free precession (FAIR TrueFISP) ASL sequence was performed in twenty-two individuals, including eighteen patients with suspected breast tumors and four healthy controls, in addition to the routine clinical imaging protocol. The definitive diagnosis was obtained by histology after biopsy or surgery. The results showed that ASL perfusion was successfully acquired in thirteen of eighteen tumor patients and all healthy controls. The mean ASL perfusion of invasive ductal carcinoma tissue was significantly higher than the perfusion of the normal breast parenchyma and invasive lobular carcinoma. No significant difference was found between the mean ASL perfusion of the normal breast parenchyma and invasive lobular carcinoma tissue. Hence, these results indicate that ASL perfusion can differentiate malignant lesions from normal breast parenchyma as well as breast tumor types. This MRI modality may be useful to detect early changes in response to neoadjuvant chemotherapy, and the signal changes proportional to the blood flow may represent a property that allows identifying potential biological markers, and consequently, developing targeted therapies. Furthermore, image acquisition can be repeated without the concern of cumulative doses of paramagnetic contrast agent, and in patients with renal insufficiency, who may not be safely injected with contrast agents [128].

### 4.4. Blood Oxygen Level-Dependent Functional Magnetic Resonance Imaging

The blood oxygen level-dependent (BOLD) functional MRI (fMRI) provides information on changes in oxygenation in tissue to measure the hemodynamic response. The BOLD “contrast” reflects a variation in the transverse relaxation rate of tissue, and the paramagnetic effects influence it by the concentration of deoxyhemoglobin [93,129]. Generally speaking, this technique is heavily used to study cerebral activity, but it has the potential to evaluate metabolism, angiogenesis, and variations of oxygenation in tumors, as well. Indeed, in preclinical models and human tumors, it has been applied as a noninvasive method to monitor antiangiogenic therapies [129,130]. However, BOLD provides an indirect estimate of oxygen delivery and has a variable and scarce relationship with tumor tissue hypoxia, which is a significant negative prognostic factor. The ability to “map” hypoxia might help therapy planning and predicts treatment failure; thus, driving early changes of therapeutic strategy [131,132,133]. Furthermore, BOLD measurements are influenced by variations in vessel caliber, by the presence of hemorrhage and movement artifacts, which hinders its implementation as a clinical biomarker of hypoxia and its readiness to translate into clinical use [131,132].

#### Breast

The BOLD MRI method has been employed as a simple, noninvasive method to assess tumor oxygenation during a preliminary observational study. Eleven patients with locally advanced breast cancer were enrolled for preoperative neoadjuvant chemotherapy with doxorubicin and cyclophosphamide for four cycles. Of these, seven patients completed all chemotherapy treatments and underwent MRI before, during, and after chemotherapy. Breast tumor response was divided into complete response, partial, and stable response based on clinical palpation. The BOLD study applied a 6-min oxygen-breathing challenge; BOLD contrast enhancement was observed in all tumors, but patients with complete responses showed a significantly higher BOLD before the start of chemotherapy compared with both partial or stable response; furthermore, there was no significant difference between latter groups. The correlation between high BOLD response and better treatment outcome suggests that this may be an excellent, noninvasive prognostic tool for cancer management, providing early predictive information on the response to chemotherapy [134].

### 4.5. Oxygen-enhanced Magnetic Resonance Imaging

Oxygen-enhanced (OE) MRI is an alternative in vivo technique to quantify and map changes, distributions, and the extent of oxygen concentrations in tumors. In OE-MRI, the longitudinal relaxation rate is used to evaluate changes in the level of molecular oxygen dissolved in blood plasma or interstitial tissue. Therefore, the measured variations are proportional to the variation of oxygen concentration in the tissue. This method allows the noninvasive identification, quantification, and mapping of tumor hypoxia with MRI in vivo, making the technique suitable for rapid clinical translation [131,133]. Indeed, tumor hypoxia and oxygen dynamics have been correlated to aggressiveness and therapeutic resistance in many tumors [135].

#### Prostate

In a preclinical study for prostate cancer, this technique was used as a potential predictive biomarker of radiation-therapy response. Dunning R3327-AT1 prostate tumors were surgically implanted subcutaneously in the flank of adult male syngeneic Copenhagen rats; nineteen days after implantation, OE-MRI was performed before each irradiation (2Fx15 Gy). The tumors were irradiated approximately 24 h after OE-MRI experiments. Before and during radiotherapy, the anesthetized animals inhaled either air or oxygen for at least 15 min. The OE-MRI and irradiation were repeated a week later, and the tumor growth delay was determined by the time required to achieve double-time volume (VDT) and quadruple time volume (VQT). Moreover, during hypofractionated radiotherapy, the BOLD and tissue oxygen-level dependent (TOLD) contrast, and the quantitative responses of relaxation rates (R1 and R2) were applied. The semi-quantitative parameters showed a significant correlation between the average TOLD and BOLD responses for single tumors before and after the irradiation. For the R1 and R2 rates, there were no significant differences before irradiation, but there was a significant difference between air and oxygen breathing. Tumors in oxygen-breathing animals expressed a more significant growth delay than tumors in the air group during irradiation. Therefore, the inhalation of oxygen during hypofractionated radiotherapy significantly improved radiation-therapy response, and OE-MRI may be added to routine clinical MRI for patient stratification and the personalization of radiotherapy treatment planning [135].

### 4.6. Diffusion-Weighted Imaging 

Diffusion-weighted imaging (DWI) is an MRI approach used to evaluate the diffusion rate of water molecules within tissues without the use of exogenous contrast agents [14]. The diffusion of water molecules and the degree of mobility are expressed quantitatively by the apparent diffusion coefficient (ADC), which can estimate the changes in cellularity and the integrity of cell membranes [99,136,137]. In particular, DWI is suited for the characterization of the tumor and for monitoring treatment response. Indeed, the variations in the ADC can demonstrate changes in the physiology of the tumors following therapeutic interventions, and therefore, ADC may be a potential biomarker of treatment efficacy [99,118,137,138,139]. The DWI has shown its value in tumor monitoring in both preclinical cancer models and clinical patients [101,138,140,141].

Preclinical assessments of early responses to therapy have been evaluated in breast and prostate cancer models, and in lymph node sites as a potential alternative method for detecting metastatic lesions. Primary applications of DWI are tumor detection and differentiation from non-tumor tissues, differentiation of malignant from benign lesions, and monitoring and prediction of treatment responses [137]. Hence, this technique may also be a noninvasive imaging biomarker of tumor aggressiveness for better stratification of patients with poor prognosis [138,139]. Indeed, this technique is being considered the standard of care for prostate and liver cancer, in which the different ADC measurements can predict tumor aggressiveness [14,93,139,142]. Additionally, in breast and thyroid cancers, ADC is considered an imaging biomarker able to differentiate malignant and benign lesions, and it has also been shown to predict the response to neoadjuvant chemotherapy in the former histotype [101,143]. Furthermore, DWI has been evaluated as a possible alternative to PET/CT for the detection of metastatic lesions [14,93,139,142].

#### 4.6.1. Thyroid

It has been shown that DWI and ADC maps, have the potential to distinguish malignant and benign nodules in the thyroid, since this technique can assess the different cellular architectures of tumors [144]. Differences in ADC were evaluated between benign and malignant nodules, correlating ADC to cytological results by fine-needle aspiration. The study included 36 patients with thyroid gland nodules and 24 healthy patients, all of whom were examined with DWI sequences. In the nodular patient group, there were 27 cases of benign nodules and nine cases diagnosed as thyroid gland malignancy; in total, 52 benign nodules and 16 malignant nodules were examined. The ADC values were significantly different between benign and malignant nodules, and from healthy thyroid tissues in the controls. In the benign group, the ADC value of thyroid nodules was increased while in the malignant group, it was reduced; in the healthy thyroid tissue, the ADC value was within the normal range. The reduction in the ADC observed in most malignant lesions was linked to the decrease in extracellular/extravascular space due to cellular proliferation, as confirmed by cytology. These results showed that DWI provides useful and promising results on the nature of a thyroid nodule, and it may have a role in the selection of nodules that should undergo needle aspiration cytology [143].

Similarly, DWI ADC mapping was performed on 14 patients with malignant thyroid nodules diagnosed by ultrasound and verified by biopsy. In these patients, 13 nodules were malignant, as shown with biopsy evidence, and five nodules were benign. Malignant nodules had significantly lower ADC values than the benign ones, confirming previous results [145].

In a preliminary study, the employment of DWI was investigated using a readout-segmented multishot EPI (RESOLVE) sequence to differentiate between well-differentiated and undifferentiated subgroups of thyroid carcinomas. Moreover, the correlations between this technique and histopathologic data, such as the Ki-67 index and p53 expression, were evaluated. Indeed, Ki-67 represents a histopathologic parameter associated with cell proliferation, whereas p53 is considered a marker of tumor aggressiveness. Fourteen patients received preoperative MRI scans, including DWI-RESOLVE, and T1 and T2 conventional sequences before and after contrast-medium administration (gadopentetate dimeglumine). Four patients showed follicular thyroid carcinoma; four patients, papillary thyroid carcinoma; and six patients, undifferentiated thyroid carcinomas. The results showed that the mean ADC values were significantly lower in undifferentiated carcinomas compared with follicular and papillary carcinomas. A decrease in ADC mean values inversely correlated with an increase in Ki-67, while the increase in p53 expression was correlated to an increased in ADC mean values. Therefore, DWI seems able to distinguish between differentiated and undifferentiated thyroid carcinomas. This approach, once further validated, might help to preselect optimal therapeutic strategies in the presurgical phase [144].

#### 4.6.2. Breast

The DWI and magnetic resonance spectroscopy (MRS) were able to detect early tumor responses in triple-negative breast cancer (TNBC) mouse xenografts after combination therapy with TRA-8, a monoclonal antibody targeted to the apoptosis receptor, and carboplatin, a standard chemotherapeutic agent. The therapeutic efficacy was assessed by monitoring tumor volume, first ADC changes, and lipid concentration through the fat–water ratio (FWR) MRS. Two different TNBC xenograft models, implanted either with 2LMP or SUM159 cell lines, were treated intraperitoneally with either carboplatin, TRA-8, or their combination. The MRI acquisitions with DWI and MRS were performed before, during, and at the end of the therapeutic protocol. Combination therapy with TRA-8 and carboplatin showed significantly reduced tumor growth in both 2LMP and SUM159 TNBC models compared to both single-drug treatments, and the therapeutic efficacy was verified histologically, as witnessed by a significant increase of apoptotic cell density. Substantial changes in ADC were detected only three days after the initiation of TRA-8 or combination therapy, while significant changes in FWR required seven days for detection. Both ADC and FWR changes were confirmed as useful imaging biomarkers to evaluate the therapeutic efficacy, but ADC changes may be used as an earlier predictor. This imaging protocol might be translated into clinical trials testing these or similar drugs, since early assessment is useful for preventing unnecessary/ineffective treatments and, hence, to improve outcomes [146].

In the preclinical model of ductal breast cancer with BT474 cells previously described (see 4.1 T1 and T2 Weighted Sequences, [109]), ADC was also tested as a predictor of gefitinib efficacy. Tumor ADC increased in all treated groups. Therefore, as mentioned, the parameters included in the study, specifically *Kps* and *fPV* from DCE and ADC from DWI, may all provide, in combination, a more sensitive approach to determining the right treatment plan [109].

A mouse model of a breast cancer brain metastasis was developed to optimize a longitudinal MRI and MRS method for analyzing the pathogenic aspects of brain metastasis, since cerebral metastasis showed a high incidence in breast carcinoma patients. Brain metastases were generated by the intra-carotid injection of human mammary carcinoma cells 435-Br1, a brain metastasis variant of the parental cell line MDA-MB-435. Different MRI approaches were used to characterize the morphological and metabolic development patterns of brain metastases, including T2 and contrast enhanced-T1 weighted images, DWI, and MRS imaging. The acquired data were then combined with the histological analyses of the dissected brains. As a result, nine out of thirteen mice developed MR-detectable abnormal masses (single or multiple) in the brain parenchyma within 20 to 62 days after injection. Two additional animals had brain metastases detected only by histological analysis. The ADC maps correctly differentiated the edema and disorders of cerebrospinal fluid circulation areas from metastases, in addition to the MRS results that have been discussed in the dedicated paragraph. This imaging approach might be considered helpful in early detection and differentiation of brain metastases from other cerebral abnormalities [147].

In the preliminary study of breast cancer in human patients already described (see 4.2 Dynamic Contrast-Enhanced [122]), the DWI method was used to predict tumors’ responses following neoadjuvant therapy (NAT), in conjunction with DCE. After completion of NAT, the pathologic complete response (pCR) and nonresponse (non-pCR) were determined at the time of surgery. The mean ADC was increased between the first two time points for the same pCR, while no noticeable change in mean ADC was observed for the non-pCR patient. The MRI approach described may improve the accuracy of evaluating the tumor response to NAT, showing a higher predictive power than models based on tumor size changes, and it may be used as an early marker of outcome in breast cancer patients [122].

#### 4.6.3. Prostate

In a prostate cancer xenograft mice model, DWI was tested as a predictor of the efficacy of docetaxel, a cytostatic and cytotoxic antineoplastic drug. The LnCaP cell line, derived from the lymph node metastasis of a human prostatic adenocarcinoma was used, since it secretes the prostate-specific antigen (PSA). Serum PSA levels and tumor volumes were used to measure tumor response noninvasively. Results showed a good response of the xenograft to docetaxel, and serum PSA was confirmed as a useful biomarker of therapy response, but changes in ADC represented an earlier indicator of therapeutic response. This approach should be validated in the clinic to correctly manage the therapeutic regimen, especially for second-line therapies that show reduced response rates and higher toxicity [148].

In the study described before (see 4.2 Dynamic Contrast-Enhanced [123]), mice bearing prostate cancer xenografts underwent, as mentioned, DWI acquisitions pre-radiation, and on day one and nine after. The ADC values, volumes, and PSA levels revealed significant correlations with treatment response. The combination of all parameters, including the *K^trans^* obtained with the DCE, successfully predicted treatment response with a high correlation coefficient. Therefore, the combination of such parameters with standard clinical parameters may improve the predictive power for the therapeutic outcome in prostate cancer patients, and it might be applied for the personalization of therapeutic approaches [123].

A multiparametric analysis to evaluate in vivo vascularization, metabolism, and physiological characteristics that are permissive for the occurrence of metastases has been tested in a prostate cancer xenograft models using noninvasive MRI and MRS. A human prostate cancer xenograft model implanted s.c. or orthotopically in the prostate was used. The tumors were derived from PC-3 human prostate cells inoculated s.c. in the right flank of SCID mice; the intact tumor tissue obtained from subcutaneous tumors was harvested and re-implanted s.c. in the flank or orthotopically with a microsurgical method of SCID mice. These mouse models underwent MRI acquisitions, including T1-weighted with the administration of albumin-Gd-DTPA, multislice DWI for evaluation of vasculature, and MRS for metabolic maps of total choline and lactate/lipid and extracellular pH (pHe). As the results showed, the tumors in the orthotopic site were identifiable by the hyperintense signal detected in DWI compared to the s.c., and they showed higher vascular volume and higher permeability, as well. In the metastases’ characterizations, several lung nodules were observed in mice with orthotopically-implanted tumors, and only a few small clusters of cells were observed in the lungs of mice with xenografts. Therefore, the higher metastatization rate of orthotopic tumors might be linked to the higher vascular volume and permeability. These data confirm the profound influence of the tumor microenvironment on the metastatization process and the feasibility of identifying noninvasive clinically translatable parameters that may contribute to determining risk factors for metastases formation in patients. Future translational studies are necessary to validate these observations further [149].

### 4.7. Diffusion Kurtosis Imaging

Diffusion kurtosis imaging (DKI) has been applied to many body-districts, showing a higher capability in differentiating between benign and malignant tumors, and in predicting treatment responses compared to conventional DWI. From the DKI model, the diffusion coefficient (D) and the diffusional kurtosis (K) are derived, which provide more information on the tissue structure than DWI. Indeed, the parameter D evaluates the tissue diffusion and seems to be more accurate than ADC; the K parameter seems to be more sensitive to irregular and heterogeneous cellular environments, but less sensitive to cell density.

#### Thyroid

In a preliminary study, the usefulness of DKI compared to DWI was investigated in thyroid lesions, also assessing the correlation of such MRI parameters with histopathologic features. Fifty-eight patients with thyroid nodules detected by ultrasound underwent MRI examination, including T1 and T2-weighted imaging, conventional DWI, and DKI. Histopathological analysis was performed with the evaluation of Ki-67 and vascular endothelial growth factor (VEGF), since Ki-67 is a cell proliferation protein and is considered a neoplastic marker, as mentioned above, and the VEGF is a cytokine that induces angiogenesis and is related to angiogenesis in tumors. Fifty-eight thyroid lesions were found, including twenty-four papillary thyroid cancers, twenty-one adenomas, seven nodular goiters, and six cases of Hashimoto’s thyroiditis. Malignant lesions showed significantly higher mean ADC and D values and lower K values compare to benign lesions. The number of positively-stained VEGF and Ki-67 cells was significantly higher in the malignant group. The DKI-derived D parameter showed the strongest correlation with both Ki-67 and VEGF. Therefore, DKI should be considered advantageous over the conventional DWI for the diagnosis of thyroid lesions with better diagnostic accuracy [150].

### 4.8. Magnetic Resonance Spectroscopy

Magnetic resonance spectroscopy (MRS) allows the identification of different atomic nuclei, such as hydrogen, carbon, phosphorus, and fluorine, in specific regions of interest; thus, providing functional and biochemical information on a wide range of biological processes. For example, among the cellular metabolites measured with MRS, there is choline contained in cell membranes, creatine and glucose involved in energy production, and alanine and lactate that are typically increased in some tumors [83]. In clinical applications, it is a noninvasive method for the assessment of tumor biochemistry and physiology to identify the early changes in response to therapy, as well as for grading and staging of cancer [93,151,152]. In many preclinical studies, e.g., in breast or colon cancer, this method is used to track intra-tumor changes during therapy, and the levels of the metabolites measured may represent a potential noninvasive marker to check tumor response [153,154,155].

#### 4.8.1. Thyroid

Proton (H) MRS and DWI-ADC mapping (see 4.6 Diffusion-Weighted Imaging, [145]) were performed on 14 patients with malignant thyroid nodules diagnosed by ultrasonography and verified by biopsy. In these patients, 13 nodules were malignant with biopsy evidence, and five nodules were benign. Intra-nodular choline (Cho) peak, Cho/creatine (Cr) ratio, and ADC values of malignant nodules were evaluated and compared to those of benign nodules. Malignant nodules had significantly higher Cho peak amplitudes and a higher Cho/Cr ratio than benign ones. Hence, the use of H-MRS may be added to other imaging approaches in the evaluation of thyroid nodules and the determination of their nature. It might help to establish adequate treatments at an early stage, reducing the morbidity and mortality of the disease, and, at the same time, avoiding unnecessary surgical interventions in patients with benign lesions. Nonetheless, there are technical difficulties in performing MRS on thyroid glands, or other neck structures, concerning tumor movement due to swallowing and breathing, substantial differences in magnetic susceptibility between the neck and the air in the trachea, and the contamination of spectra by adjacent fat [145].

#### 4.8.2. Breast

In the TNBC xenograft mouse models previously described (see 4.6 Diffusion-Weighted Imaging, [146]), MRS was associated with DWI to detect early tumor response after combination therapy with TRA-8 and carboplatin. The therapeutic efficacy was assessed by monitoring tumor volume, ADC changes, and lipid concentration through the fat–water ratio (FWR) MRS. The MRI acquisitions with DWI and MRS were performed before, during, and at the end of the therapeutic protocol. Significant changes in FWR required seven days for detection. Both ADC and FWR changes were confirmed as useful imaging biomarkers to evaluate the therapeutic efficacy, but ADC changes were detected earlier than FWR. This imaging protocol should be translated into clinical trials to improve outcomes by stopping ineffective therapies [146].

In the brain metastasis model previously described (see 4.6 Diffusion-Weighted Imaging, [147]), MRS was again added to DWI. A single-voxel MRS was used to analyze the metabolic patterns of the lesions in ten subjects. Only eight out of ten mice had brain metastases, and just two of them showed a field homogeneity good enough and a correct voxel position inside the lesion. However, when the lesions increased in size and infiltrated the brain parenchyma, the spectral changes indicated the replacement of the healthy tissue pattern with the tumor tissue pattern. The data proved a decrease in N-acetyl aspartate (NAA) as the earliest sign of metastasis growth, followed by a decrease in Cho and Cr levels. Those results suggest that this approach, in addition to DWI, may help in discriminating brain metastasis growth and the classification of distinct progression stages, and it might open the way to its use in the diagnosis and therapy monitoring [147].

Phosphorus (^31^P) MRS was used to quantify the levels of phosphorylated metabolites in breast cancer tissue. In particular, phosphocholine (PCho), glycerophosphocholine (GPC), phosphoethanolamine (PEtn), and glycerophosphoethanolamine (GPE) may be considered valuable biomarkers for the diagnosis and noninvasive monitoring of therapies both in preclinical studies and in the clinical phase. To this end, the metabolic response to the oral treatment with phosphatidylinositol-3-kinase/mammalian target of the rapamycin (PI3K/mTOR) inhibitor BEZ235 was evaluated in basal-like (MAS98.12) and luminal-like (MAS98.06) breast xenograft models. These models were established by direct implantation of primary human breast cancer tissues in the mammary fat pad of immunodeficient mice, and then serially transplanted in BalbC nu/nu mice. As a result, a significant increase in GPC and PCho was found in basal-like xenografts, whereas PEtn decreased. No significant changes were observed in phosphorylated metabolites in luminal-like xenografts, which did not respond to treatment with BEZ235. These data demonstrate the usefulness of 31P MRS in the metabolic profiling of breast cancer subtypes and the evaluation of the metabolic response to targeted anticancer drugs [156].

In a pilot study, the use of MRS was tested for monitoring breast cancer therapy. Indeed, such neoplastic tissue contains high levels of choline-containing compounds (tCho). The changes in tCho concentration were determined to predict the early clinical response to neoadjuvant chemotherapy in the first 24 h after initial treatment in patients with locally advanced breast cancer. Sixteen women, aged 18 to 80 years, with biopsy-confirmed, locally advanced breast cancer, were enrolled. Thirteen patients received combined doxorubicin hydrochloride and cyclophosphamide treatment that was administered on day one, with additional doses at 21-day intervals for a total of 64 days. Patients underwent MRS before treatment, within 24 h after the first dose, and then after the fourth dose. As a result, eight out of thirteen patients showed a significant correlation between changes in tCho concentration and lesion-size reduction. These patients had a lower tCho level within 24 h after the first dose compared to baseline and a further decrease in the tCho concentration after the fourth dose of the combined therapy. The other five patients out of the thirteen showed no changes and had a baseline tCho concentration less than or equal to that measured within 24 h after the first dose. Therefore, MRS may be used to assess early response to neoadjuvant chemotherapy and to customize an effective regimen for individual patients [157].

#### 4.8.3. Prostate

In the prostate cancer model described (see 4.6 Diffusion-Weighted Imaging, [148]), MRS was applied to obtain metabolic maps of total choline and lactate/lipid ratio, and extracellular pH (pHe). Total choline and lactate/lipid levels were significantly higher in orthotopic compared with xenograft tumors, whereas the pHe maps showed a significantly lower pH in orthotopic tumors compared with subcutaneous tumors. As already stated, such an imaging approach may prove to be extremely helpful in studying the tumor microenvironment, demonstrating changes that might be linked the metastatization process, like in this case, total choline and a more acidic extracellular pH. Once validated, these techniques may also support the development of novel strategies to reduce metastatization [148].

### 4.9. Chemical Exchange Saturation Transfer

Chemical exchange saturation transfer (CEST) is an MRI method to detect low concentrations of metabolites for probing specific molecular and physiological events. The sensitivity of this approach is enhanced by the use of a set of new specific contrast agents, and endogenous as well as exogenous molecules can be used. Indeed, a variety of molecules have been demonstrated as potential contrast agents in this technique, including small diamagnetic molecules, paramagnetic ions complexes, liposomes, nanoparticles, and hyperpolarized gases [158,159,160]. The CEST applications in the clinic aim to monitor different metabolites, such as glycogen concentration (glycoCEST), glycosaminoglycans levels (gagCEST), or glutamate (gluCEST). Moreover, this is a valid imaging modality for detecting and monitoring the progression of tumors and assessing their responses to therapy by avoiding exposing the patient to radiation [159,161,162]. In preclinical studies, CEST has been used to measure the rate of metabolites’ uptakes, such as glycogen and glucose, which are hallmarks of the tumor microenvironment. The evaluation of extracellular pH (acidoCEST), which is linked to the increase in lactic acid production after an increase in glycolysis, may be used for assessing tumor aggressiveness and early responses to treatments that inhibit glycolytic metabolism [161,163,164]. The CEST measurement of glucose metabolism (glucoCEST) in tumor mouse models allows studying the tumor microenvironment, and it may represent a potential replacement of the PET approach [159,164].

#### 4.9.1. Breast

The CEST has been proposed as a new molecular imaging approach to detect glucose or its analogs in the diagnosis of tumors. D-glucose and deoxy-D-glucose (2DG) were commonly employed to that end, but their toxicity at high concentrations precludes their clinical use and limits preclinical applications. A preliminary experiment was conducted to examine the validity of 3-O-methyl-D-glucose (3OMG) as a nontoxic alternative, which has been demonstrated to be able to detect tumors in several models of murine and human breast cancer. Moreover, this method was compared with glucoCEST and [18F]-FDG PET on the same animals. Orthotropic tumors were induced in mice by injecting human MDA-MB-231 or MCF7 cells. The CEST MRI sequences were performed on mice before and after the administration (intravenous, intraperitoneal, or oral) of 3OM. The same animals were then injected with [18F]-FDG for PET imaging, and D-glucose for glucoCEST after a specific time. The results showed that the CEST MRI following the administration of 3OMG produced patterns that reflected the metabolic activity of tumors and clearly distinguished them from other body districts. A marked 3OMG-CEST MRI contrast was obtained, and the most aggressive breast cancer models produced the highest CEST contrast. The contrast reached its maximum at 20 min post administration and lasted for more than one hour, without any difference in effect levels or timing between the three routes of administration. The 3OMG CEST method compared to the glucoCEST showed a higher CEST contrast than D-glucose. Moreover, a good correlation was found between 3OMG CEST contrast and FDG uptake, providing clear validation of this technique. Therefore, the validation of the 3OMG-CEST MRI method in the clinic would offer significant advantages for evaluation, detection, and monitoring of tumors’ progressions, and assessing their responses to therapy, avoiding radiation exposure [161].

#### 4.9.2. Prostate

The CEST approach, using amide proton transfer (APT) MR imaging, was used in a preliminary study to localize prostate cancer better and to detect the difference in cancer aggressiveness, discriminating between cancerous and non-cancerous tissues. The APT MRI does not require the injection of a contrast agent since it uses endogenous amide protons in tissue, which allows detecting micromolar concentrations of mobile proteins with high sensitivity. Therefore, the applicability of this imaging modality to the detection of prostate cancer was based on the high rate of tumor cell proliferation and on the cellular density of this tumor, which leads to high levels of mobile proteins. In this study, twelve patients with biopsy-proven prostate cancer scheduled for prostatectomy were enrolled and underwent T2 and APT MRI acquisitions. The APT ratio in the tumor zone was significantly higher than that in the benign regions of the peripheral zone; hence, distinguishing them. Such results were confirmed by both the T2-weighted imaging and histopathological findings. The CEST APT imaging technique may, thus, represent a potential approach to detecting and discriminating between low and high-grade prostate cancer, and it might be more specific compared with DCE or DWI sequences [164].

### 4.10. Short Tau Inversion Recovery

The short tau inversion recovery (STIR), is an inversion recovery sequence, which is a spin-echo sequence with a 180° preparation pulse to flip the longitudinal magnetization into the opposite direction. In this case, to generate an MR signal, the longitudinal magnetization is then converted to transverse magnetization through the application of a 90° pulse. The time between the change from 180° to 90°, or inversion time in such sequences, is kept short, between 130 and 150 ms.

#### Breast

In a preliminary clinical experience, the usefulness of whole-body turbo STIR sequence was assessed to detect liver, brain, and bone metastases as a single examination in breast cancer patients, in alternative to conventional techniques. Seventeen patients with biopsy-proven breast cancer and suspected metastatic disease were included in this study and underwent both whole-body STIR-MRI and conventional imaging, such as MR brain imaging with spin-echo; T1 and T2-weighted CT and ultrasound scanning; and bone scintigraphy. Three patients were found to be free of metastases in both conventional and STIR imaging. In eleven out of seventeen patients, appendicular or axial skeletal metastases were identified, with a good correlation between findings in whole-body STIR and scintigraphy. Hepatic metastases were found in five patients using whole-body STIR, of which only three patients’ finding correlated with CT and ultrasound findings. Metastases and brain abnormalities were found in four patients via both whole-body STIR and dedicated brain MRI. Therefore, this pilot evaluation, even if performed on a small cohort of patients, showed that STIR acquisition might represent an accurate, convenient, and cost-effective staging method during the long-term follow-up of breast cancer patients [165].

## 5. Conclusions

Translational research must be supported by a real interdisciplinary, synergistic collaboration to avoid the inconveniences and ineffectiveness of the methods, in order to emphasize the benefits of patients in a robust translational application. In order for an experimental imaging methodology to be successfully translated into clinical practice, it must have a substantial and immediate impact on application to the patient, and therefore, be easily accessible. The development and use of animal models of cancer and its integration with molecular imaging tools may enhance the clinical translatability of preclinical studies focused on the clinical implementation of MRI methodologies in the management of tumor diseases from the diagnoses to therapeutics’ evaluations and follow-up monitoring. From that perspective, it should be borne in mind that there is not a single ideal mouse model that recapitulates all features of the human pathology. Hence, the development and choice of the appropriate model must comply with the experimental, or from a translational perspective, with the direct clinical needs. Thus, it is fundamental to evaluating which the characteristics that may influence clinical efficacy are; i.e., histopathologic features, immune system interaction, carcinogenesis, angiogenesis, tumor progression, and metastatic potential. Indeed, as described, each model has its peculiarities, and all might be considered complementary to each other. Finally, the best combination of the appropriate experimental model and the careful choice of imaging modalities with clinical homologs might accelerate the reduction in the gap between preclinical and clinical studies. The high translational ability of MRI, among the other imaging techniques, due to the high similarity in terms of hardware and software between experimental and clinical scanners, should strengthen the efforts in evaluating how far this technique can go for the diagnosis and management of cancer patients.
